# Drug Use during Acute Illness in Tigray Region, Northern Ethiopia: A Household Study

**DOI:** 10.1371/journal.pone.0145007

**Published:** 2015-12-14

**Authors:** Abrham Wondimu, Fantahun Molla, Solomon Abrha, Jemal Mohammed, Birhanu Demeke, Tadele Eticha, Admassu Assen, Wondim Melkam, Naod Gebre-Samuel, Derbew Fikadu Berhe, Ebisa Tadese, Kedir Endris

**Affiliations:** 1 Department of Pharmacy, College of Health Sciences, Mekelle University, Mekelle, Ethiopia; 2 Department of Nursing, College of Health Sciences, Mekelle University, Mekelle, Ethiopia; Nottingham University, UNITED KINGDOM

## Abstract

**Background:**

Drug use study in the community enables health authorities to understand pattern of drug utilization and its related aspects. This, in turn, can help to develop rational drug policies to be harmonized in accordance to the need of the community.

**Objective:**

The aim of this study was to assess drug use during acute illness by the general population in Tigray region, Northern Ethiopia.

**Method:**

A community based cross-sectional study was undertaken in April 2013 in Tigray Region, Ethiopia. A total of 1034 households were interviewed in the study. A multi-stage sampling technique was used to select households. Data were collected using a pre-tested structured questionnaire. Data were analyzed using descriptive statistics and bivariate and multivariate logistic regression model.

**Results:**

Out of 1000 households, 210(21%) reported an episode of acute illness. The prevalence of acute illnesses in rural areas 126(25%) (AOR = 1.83, 95% CI: 1.21–2.76) was significantly higher than that of urban areas 84(17%). Cough, runny nose, sore throat, earache, fever and headache added up to 155(52%) of all reported symptoms of acute illnesses. The majority of the patients 162 (77%) took modern medications for the managements of their diseases. Half 105(50%) of the consumed medications were antibiotics. The large proportions 173(83%) of medicines for acute illness were taken orally. The greater proportions 150(93%) of medications were prescribed by health professionals. Thirty-four households (21%) reported treatment discontinuation.

**Conclusion:**

The prevalence of acute illnesses in this study was found to be 21%. Acute illnesses were more common in rural areas than urban areas. Antibiotics were the most frequently used drugs for acute illnesses.

## Introduction

The importance of modern therapeutic agents for diagnostic and curative purposes and their contribution to health require no emphasis. A remarkable increase in the use of drugs is seen in almost every country. Modern medicaments, however, tend not only to be expensive and to add appreciably to the cost of health services but they can also be harmful in certain circumstances [1]. Hence, it is found mandatory to conduct studies that focus on the process of drug utilization and factors related to prescribing, dispensing, administering and taking of medication [2].

A significant number of studies have been conducted in various developed and developing countries. In contrast to a relatively large amount of studies in hospitals [2, 3, 4], there are few community-based data describing the pattern of drug usage in the general population in different developing countries particularly in Ethiopia. To that end, the studies become rare and incomplete when community based studies for acute illnesses are emphasized. This lack of studies in the field, where medicines are actually consumed, prevents us from understanding the problems perceived by consumers and hampers the development of adequate policies. In general, in order to develop rational policies concerning drug use, information must be obtained about the local context of drug distribution, and must come from the dispenser's as well as the consumer's perspective [5]. Therefore, the present study was designed to assess the drug use during acute illness by the general population in Tigray region, Northern Ethiopia.

## Methods

### Study area and design

A community based cross-sectional study was conducted in Tigray Regional state, Northern Ethiopia in April 2013. Administratively, Tigray regional state is structured into seven zones: Eastern Tigray, Western Tigray, Northwestern Tigray, Southern Tigray, Southeastern Tigray, Central Tigray, and Mekelle. Mekelle is the capital city of Tigray Regional state, and is located 783 kilometers north of Addis Ababa, the capital city of Ethiopia. According to the 2007 Census conducted by the Central Statistical Agency of Ethiopia (CSA), Tigray Region has an estimated total population of 4,314,456 and households of 985,654 with an average of 4.4 persons per household [6].

### Sample size determination and sampling

Sample size was determined using a two population proportions formula based on the prevalence of medicine users in urban area 74% [7] and rural area 61.5% [8]. The sample size was calculated to be 1034, by taking β = 0.2 and 5% margin of error at 95% confidence level. Design effect of 2 and 5% non-response rate were also taken into consideration while calculating the sample size.

Multi-stage sampling technique was employed to select the study participants. First, two Zones (Southern Tigray and Eastern Tigray Zones) were selected from the Tigray Regional state by simple random sampling technique (using a lottery method) in view of that the seven zones in the region were homogeneous. Second, one urban administrative town (urban district) and one rural district were selected randomly from each of the selected Zones. With the help of knowledgeable community leaders, a rough map of the settlement was drawn using basic landmarks, such as roads, streams, churches and schools. Finally, numbered lists of all households were made for each cluster. Then, a random number table and proportional to size allocation techniques were used to obtain the total sample size of n = 1034.

### Data collection and analysis

Data were collected by trained health professionals (pharmacists) using a structured questionnaire interview adopted from other similar studies (S1 File). The questionnaire was translated into local language, Tigrigna and back into English to maintain its consistency by independent language experts. Data were collected from the household head or any adult member (≥18 years) present at home during the time of data collection. The overall data collection activity was supervised by the investigators. The collected data were coded and checked for completeness and consistency. Data were entered using Epi-Data version 3.1 and analyzed using SPSS version 20.0 statistical software. The data were summarized by descriptive statistics using frequency and percentage distribution and bivariate logistic regression analysis was performed to compare urban and rural areas. Significant factors in bivariate analysis were considered for multivariate logistic regression model.

### Inclusion criteria

Households in the study area in which the head of household or any adult member (≥18 years) was present, willing and capable to provide data were included in the study.

### Ethical Considerations

The study protocol was reviewed and approved by Health Research Ethics Review Committee of College of Health Sciences, Mekelle University. Official letter of cooperation was obtained from Tigray Regional Health Bureau and was distributed to respective community leaders. Permission was obtained from the community leaders. The respondents were briefed about the aim of the study and written informed consent was obtained before the data collection. Confidentiality and privacy were maintained during data collection. Moreover, no personal identifier was taken and each questionnaire was coded.

### Operational definitions

An acute illness is a condition that appears suddenly; the person did not have it immediately before becoming ill.

Modern medicine is western or non-traditional medicine.

## Results

### Socio-demographic characteristics of the respondents by acute illness

Out of 1034 households, 1000 households were interviewed which gives a response rate of 97%. Five hundred four (50%) were urban households and 496(50%) were rural households, and the median family size of the households was 5 (IQR = 3). Eighty four (17%) urban and 126 (25%) rural households reported at least one acute illness during the data collection in four weeks of recall period. Results from the multivariate logistic regression analysis indicated that the occurrence of acute illnesses in rural area (AOR = 1.83, 95% CI: 1.21–2.76) was significantly higher than urban area. Of the households reported some kind of acute illness, nearly half (48%) had at least one child less than five years old and about three in five (59%) had mothers who were illiterate as a member of the family (Table 1).

**Table 1:** Socio-demographic characteristics of households by acute illness in Tigray region, Northern Ethiopia, April 2013.

Characteristics		Having acute illness	
		Yes, n (%)	No, n (%)
**Residence**	Urban	84 (17)	420 (83)
	Rural	126 (25)	370 (75)
**Family size**	<5	96 (20)	388 (80)
	≥5	114 (22)	402 (78)
**Number of children less than 5 years**	0	109 (20)	441 (80)
	1	76 (23)	261 (77)
	≥2	25 (22)	88 (78)
**Father’s education level**	Illiterate	54 (29)	134 (71)
	Read & write	44 (20)	175 (80)
	Primary education	20 (16)	105 (84)
	Secondary education	18 (18)	83 (82)
	Tertiary education	18 (17)	87 (83)
**Mother’s education level**	Illiterate	121 (25)	369 (75)
	Read & write	28 (17)	137 (83)
	Primary education	19 (15)	110 (85)
	Secondary education	17 (17)	83 (83)
	Tertiary education	20 (23)	67 (77)
**Presence of health professional in the family**	No	198 (21)	735 (79)
	Yes	12 (18)	55 (82)

### Acute illness characteristics

Two hundred ten (21%) households reported acute illness in the four weeks of recall period and 170(81%) of them reported that one person in the household had acute illness. Type of acute illness symptoms reported by the households is shown in Table 2. The major groups of symptoms reported were Cough, runny nose, sore throat or earache 92 (32%).

**Table 2:** Symptoms of acute illnesses reported by the households in Tigray, Northern Ethiopia, April 2013.

Conditions	Frequency (N = 286)	Percentage
Cough, runny nose, sore throat, ear ache	92	32
Fever, headache	63	20
Diarrhea, vomiting, nausea	63	20
Pain, aches	29	10
Bleeding, burn, accident	16	6
Difficulty breathing, fast breathing	16	6
Others*	7	3

* Convulsions, Could not sleep, Thirst, sweating

### Medications use practice

**Fig 1:**
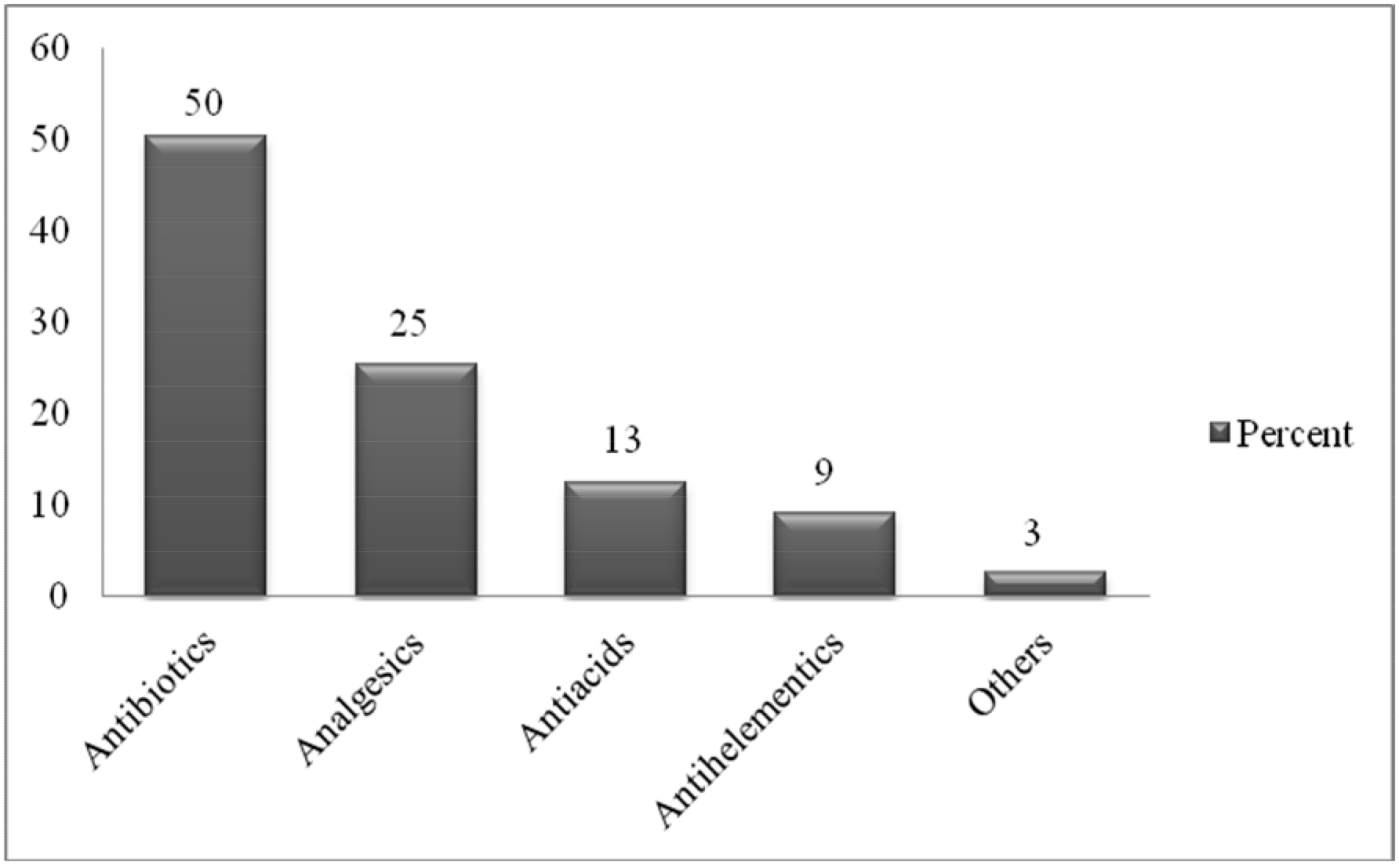
Categories of medicines used for acute illness. Fig 1 indicates categories of medicines used for acute illness as reported by the households in Tigray region, northern Ethiopia.

**Fig 2:**
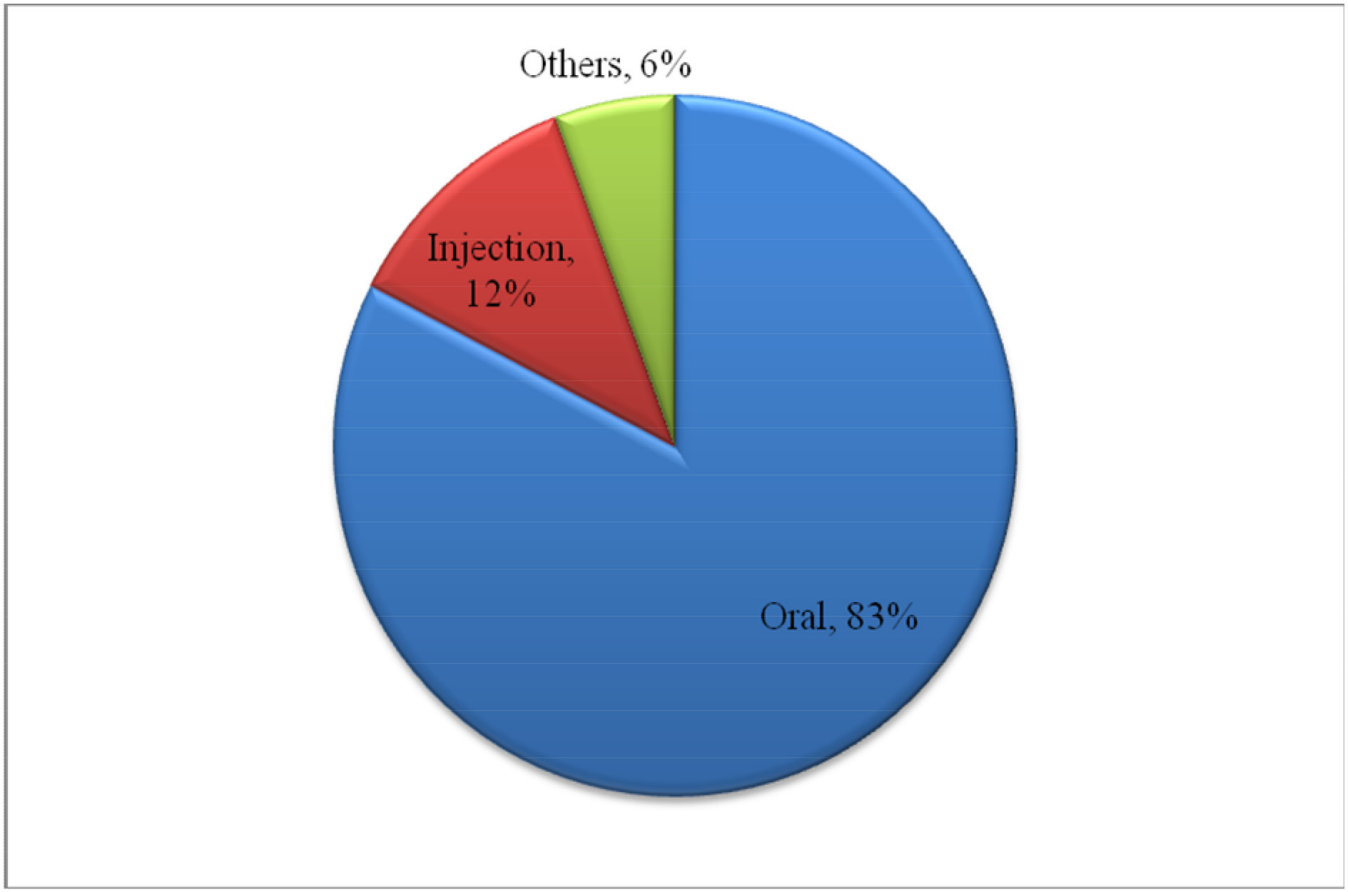
Route of administration of medicines for acute illness in Tigray region, Northern Ethiopia, April 2013. Fig 2 shows routes of administration of medications kept at home for acute illness in Tigray region, northern Ethiopia.

One hundred sixty two patients (77%) with acute illness took modern medications for the managements of their diseases. Fig 1 shows categories of medicines used for acute illness as reported by the households. Antibiotics 105(50%) were the most frequently used drug category. The large proportions of medicines for acute illness were taken orally 173(83%) (Fig 2). Distribution of drug prescribers for acute illness and their sources of supply are given in Table 3. About fifty sixth (84%) of the drugs were prescribed by physicians/nurses and almost all 157(97%) of these drugs were obtained from health facilities. Thirty four 34(21%) of the people with acute illness discontinued their medication. The main reason for not taking medication as recommended was starting to feel better which was cited by 11 (28%); followed by the belief that the drug is not working 7 (18%) (Table 4).

**Table 3:** Distribution of drug recommenders/prescribers for acute illness and their sources.

Variable	Frequency	Percentage
**Recommender/prescriber**		
Physician/nurse	136	84.0
Pharmacist/druggist	14	9
Friend/neighbor	4	3
Self	5	3
Others	3	2
**Sources of medicines**		
Public hospital	64	40
Public health center	59	36
Private pharmacy/drug store	24	15
Private health center	10	6
Others	5	3

**Table 4:** Reasons for discontinuing the medications in Tigray region, Northern Ethiopia, April 2013.

Variable	Frequency	Percentage
People discontinued their medications	34	21
**Reasons for discontinuing the medications**		
Started to feel better	11	28
Believe the drug is not working	7	18
Due to the side effect	5	13
To save for later use	2	5
Others	14	36

## Discussion

This household based survey revealed different aspects of drug utilization at household level including sources for acquisition of drug products, common reasons for consumers’ request for drugs, common therapeutic categories of drugs used during acute illnesses in a population of rural and urban communities, and reasons for discontinuing medications.

Within the recall period of four weeks, 210(21%) households reported an episode of acute illness in at least one member of their family members. The prevalence of acute illnesses among rural households 126(25%) was significantly higher as compared to urban households 84(17%). Poor living conditions and habits in rural communities could be the reason for increased episodes of acute illnesses in rural household. Moreover, the possible explanation for the high prevalence of acute illnesses in rural area could be related to less educated fathers and mothers, and lower number of family members working in the health facilities. The results of the present study were consistent with findings of other study conducted in Ethiopia where there was a higher prevalence of illnesses among rural residents (24%) than Addis Ababa residents with better socioeconomic status [9].

Looking at reported symptoms of acute illnesses, cough, runny nose, sore throat, earache, fever and headache added up to 52% of all reported symptoms of acute illnesses. This reflects a high prevalence of acute respiratory illnesses among rural and urban residents in Ethiopia. These findings were supported by a study conducted to analyze the practice of self-medication practice in three towns in Northwestern Ethiopia where symptoms of respiratory illnesses contributed to just over 40% of all illnesses [10]. Similar findings were reported in different studies elsewhere [11, 12, 13].

The most commonly consumed medications during reported cases of acute illnesses were antibiotics (50%) in this study. The prevalence of antibiotics use in the study area is high like another study [14]. It has been established that most cases of acute respiratory manifestations are of viral causes for which antibiotic use (anti-bacterial drugs in these cases) is discouraged. Therefore, the use of antibiotics in outpatient as well as inpatient setups must follow accepted guideline at all times in order to limit the far-reaching consequences of developing drug resistance due to inappropriate drug use.

Over-use of antibiotics, injectables and unnecessary expensive drugs are among the most common predictors of irrational drug use by prescribers and consumers [15, 16]. In the current study, lower frequency of injectable medications (18%) was reported compared to those orally administered (83%) drugs. This result is remarkably lower than the findings in different part of Ethiopia [14, 17, 18] however it is comparable with the study conducted in Ghana [19]. Considering the old beliefs in the community that injections are supposedly more effective than other dosage forms of drugs [4], the use of injectable medications for acute illness is relatively lower than several other reports. In all circumstances the use of injectables and antibiotics should be justified with therapeutic and economic considerations.

The principles of rational drug use underline that the right drug/ group of drugs must be used for the right disease condition and the right category of patients among many other such considerations [20]. This implies that consulting health professional before starting to take any medication is advisable. In the present study, majority (93%) of all medications consumed by household members who suffered from acute illness/es were prescribed by health professionals: physicians, pharmacists or nurses. This is finding is in corroboration with the studies done elsewhere [12, 13, 19].

The current study found that almost all of the drugs (97%) were acquired from health facilities. This finding might indicate behavioral changes that came through time when it is compared to data from a study conducted in Addis Ababa in between 1995 and 1996 where 156 out of 903 respondents (17%) practiced drug sharing [7]. While it is encouraging to learn that most of the family members included in this study had access to health facilities to consult and acquire medications during acute illness, it is also imperative to focus on discouraging sharing drugs among family members and neighbors.

As one of the principles of rational drug use dictates patients are normally required to take their medications for the recommended period by prescribers and/ or dispensers. Thirty four households (21%) reported that there was discontinuation of a medication regimen once started for the treatment of an acute illness in this study. This result is remarkably greater compared to the data reported by Amare *et al* [7] in their study of drug use pattern among Addis Ababa community where only 6% of respondent disclosed discontinuation of treatment. Of those respondents who reported discontinuation of treatment, close to a third (28%) claimed that treatment was discontinued because of starting to feel better after sometime on treatment. This finding is strikingly smaller compared to the value reported by a WHO survey on pharmaceutical use in Philippines [12] where 83 out of 110 (71%) of respondents discontinued their treatment for acute illness just because they started to feel better. Medication non-adherence has been a major problem globally. Several factors contribute for patients’ non-adherence to prescribed therapeutic regimens. These include unbearable side effects, symptom free conditions, lack of access and unaffordability of drugs and other personal and socioeconomic reasons [21]. Looking the fact that the majority of the drugs prescribed in this study were antibiotics, the issue of treatment discontinuation become a great concern as our world is challenged with antibiotic resistance in recent days. Therefore, appropriate education campaign has to be in place to encourage people to stick to their regimen schedule.

## Conclusion

The prevalence of acute illnesses in Tigray region was 21%. Acute illnesses were more common in rural areas than urban areas. Symptoms related to acute respiratory infections were the most commonly reported illnesses. Antibiotics were the most commonly consumed medications during reported cases of acute illnesses while lower frequency of injectable medications was observed. Majority of the medications were prescribed by health professionals. Significant number of respondents discontinued their treatment. The most reported reason for treatment discontinuation was feeling better after sometime on treatment. From the preceding, it can, therefore, be recommended that appropriate education campaign has to be in place to encourage people to stick to their medication regimen.

## Supporting Information

S1 File A structured questionnaire used to collect data. (PDF)
