# Supplementary material for: Drug Use during Acute Illness in Tigray Region, Northern Ethiopia: A Household Study
Source: PLoS One. 2015 Dec 14;10(12):e0145007. doi: 10.1371/journal.pone.0145007 (PMC4685994; doi:10.1371/journal.pone.0145007)
Supplement: S1 File — (PDF) [file pone.0145007.s001.pdf]

Questionnaire Form

Respondent No.: ________________

Community:

☐ Urban ☐ Rural

Date of interview: _____________________________

Interviewers: Name: ___________________________

Name: ___________________________

Part I: Socio-demographic information

1. Number of family members_____________________
2. Number of family members < 5 years of age_________________
3. Number of family members > 65 years of age________________
4. Educational level of the father:

   ☐ Illiterate ☐ Secondary school

   ☐ Read and write only ☐ College and above

   ☐ Primary school
5. Educational level of the mother:

   ☐ Illiterate ☐ Secondary school

   ☐ Read and write only ☐ College and above

   ☐ Primary school
6. Is there any member in the family working in a health sector?

   ☐ Yes ☐ No

7. If yes, specify his/her Job _____________________________

Part II. Use of Medicine

1. Has anyone in the household been ill in the past four week with an acute illness? *An acute illness is a condition that appears suddenly: the person did not have it immediately before becoming ill.*

   ☐ Yes ☐ No →**If No, Skip to Question 11**

2. How many persons had an acute illness over the past four week?______________
3. What is the age of the youngest person who was ill?_____________
4. What is the sex of the youngest person who was ill?

   ☐ Male ☐ Female

5. What type of problems did this person have during the illness? *Do not read. Tick one box for each group of symptoms mentioned:*

   ☐ Cough, runny nose, sore throat, ear ache
   ☐ Difficulty breathing, fast breathing
   ☐ Fever, headache, hot body
   ☐ Convulsions, fits
   ☐ Could not sleep
   ☐ Diarrhea, vomiting, nausea, could not eat
   ☐ Thirst, sweating
   ☐ Pain, aches
   ☐ Bleeding, burn, accident
   ☐ Do not know
   ☐ Other (please specify): ______________

6. How serious do you think the illness was? *Read the choices. Tick one box.*

   ☐ Very Serious ☐ Somewhat Serious ☐ Not Serious

7. Did he/she take any medicine during the acute illness, including medicines taken during hospitalization?

   ☐ Yes ☐ No

8. Which medicines were taken during this illness? *Write one medicine per row, and use codes provided in each column to collect information about each medicine.*

	Medicine	Route	Recommended/prescribed by		Obtained from	
	Write name of medicine if not known, write the most detailed category given by respondent (antibiotic, antimaleri, for fever)	1=oral<br>2=injection<br>3=others (specify)	1 = self<br>2 = household member<br>3 = friend/neighbour<br>4 = doctor/nurse	5 = traditional healer<br>6 = druggist/ pharmacist<br>9 = other (specify)	1= family, friend, neighbor<br>2=public hospital<br>3=public health center or dispensary<br>4=private health care provider	5=traditional healer<br>6=private pharmacy<br>7=left over from previous t/t<br>7=supermarket/kiosk<br>9=others (specify ________________)
Med1						
Med2						
Med3						

9. Did the sick person take all medicines that were recommended or prescribed?

☐ Yes →**If Yes, Skip to Question 11** ☐ No

10. I am going to give you possible reasons why the sick person did not take medicines. Can you tell me whether these were reasons why?

☐ Started to feel better

☐ Did not believe all the medicines were needed

☐ Believe the drug is not working

☐ To save for later use

☐ Due to the side effect

☐ Someone in the household decided medicines were not needed

☐ Someone advised not to take medicinesSick person had bad reactions to medicines in the past

☐ Someone in the household chose a different treatment

☐ Other (please specify): _________________

መጠይቅ ቅጥዒ

ቁፅሪ መልሲ ወሃቢ ___________________________

ዝነብረሉ ማሕበረሰብ

☐ ከተማ ☐ ገፀር

ቃለ መሕተት ዝተገበረሉ ዕለት: ________________

ቃለ መሕተት ዝገበረ ሽም: ____________________

ክፍሊ ሀ ፤ ማህበረ-ስነ-ህዝባዊ መረዳእታ

1. በዝሒ ስድራ ቤት ___________
2. በዝሒ ኣባላት ስድራ ቤት ትሕቲ 5 ዓመት ዕድሜኦም ____________________
3. በዝሒ ኣባላት ስድራ ቤት ልዕሊ 65 ዓመት ዕድሜኦም __________________
4. ደረጃ ትምህርቲ ኣቦ

☐ ዘይተምሃረ ☐ ካልኣይ ደረጃ ዘጠናቐቐ

☐ ምንባብን ምፅሓፍን ጥራይ ዝኽእል ☐ ኮሌጅ ዘጠናቐቐን ካብኡ ንላዕልን

☐ ቀዳማይ ደረጃ ዘጠናቐቐ

5. ደረጃ ትምህርቲ ኣዶ

☐ ዘይተምሃረት ☐ ኻልኣይ ደረጃ ዘጠናቐቐት

☐ ምንባብን ምፅሓፍን ጥራይ እትኽእል ☐ ኮሌጅ ዘጠናቐቐትን ካብኡ ንላዕልን

☐ ቀዳማይ ደረጃ ዘጠናቐቐት

6. ካብ ስድራ ቤትኩም ውሽጢ ኣብ ጥዕና መዳይ ዝሰርሕ ሰብ ኣሎዶ?

☐ እወ ☐ የለን

7. መልሶም/ን "እወ" እንተኾይኑ ስርሑ/ሓ'ዶ ይገልፁልና?__________________________

ክፍሊ ለ፡ ኣጠቓቕማ መድሓኒት

1. ኣብተን ዝሓለፋ ኣርባዕተ ቅነታት ካብ ስድራ ቤትኩም ኣባል ኣፃዳፊ ሕማም (Acute illness) ዘጋጠሞ ሰብ'ኣሎዶ? (*ኣፃዳፊ ሕማም ማለት ሃንደበታዊ ዝርአ ሕማም ኮይኑ እቲ ዝሓመመ ሰብ ቅድሚኡ እቲ ዓይነት ሕማም ዘይተርኣዮን።*)

   ☐ እወ ☐ የለን መልሰም የለን እንተኾይኑ ቀጥታ ናብ ተ.ቁ 11 ይኺዱ

2. ኣብተን ኣርባዕተ ቅነታት ውሽጢ እቲ ኣፃዳፊ ሕማም ዝሓዞም ኣባላት ስድራ ቤትኹም በዝሖም ክንደይ ይኾኑ?_____ _________

3. ካብቶም ዝሓመሙ እቲ ዝነኣሰ ዕድመ ክንደይ እዩ?________________________

4. ናይ እቲ ዝነኣሰ ዕድመ ሕሙም ፆታ እንታይ'ዩ?

☐ ተባዕታይ ☐ ኣንስታይ

5. እቲ ዝሓመመ ሰብ እንታይ ዓይነት ናይ ጥዕና ፀገም ነይሩዎ? *ሓበሬታ ንሓታታይ፣ እቶም ቀፂሎም ዘለው ምልክታት ኣየንብቡ! ክዝርዝሩ ከለው ጥራሕ ኣብቲ ዝተጠቐሰ ምልክት ይግበሩ።*

☐ ሰዓል ፣ ቀጢን ንፋጥ ብበዝሒ፣ ምቁሳል ጉሮሮ

☐ ናይ ምትንፋስ ፀገም፣ ቀልጢፍካ ምትንፋስ (ምልህላህ)

☐ ናይ ሰውነት ሙቀት ምውሳኽ (ትኩሳት፣ ረስኒ)፣ ርእሲ ሕማም

☐ ናይ ሰውነት ምንቅጥቃጥ

☐ ድቃስ ምስኣን

☐ ተቅማጥ፣ ንላዕሊ ምባል፣ ዕውልውል ምባል፣ ምግቢ ዘይምብላዕ

☐ ፃምእ ማይ፣ ምርሃፅ

☐ ቃንዛ፣ ሕማም

☐ ምድማይ፣ ምቅፃል፣ ሃንደበታዊ ሓደጋ

☐ ኣይፈልጦን

☐ ኻልኦት ፀገማት ተሃልዮም ይዘርዝሩ፡፡________________________________

6. እቲ ሕማም ክንደየናይ ከቢድ ነይሩ? *ሓበሬታ እቶም መማረፂታት ኣንብቦም/የም።*

☐ ብጣዕሚ ኸቢድ ☐ ኸቢድ ☐ ቐሊል

7.ሓሚሙ/ማ እንዳሃለወ/ት ዝኾነ ዓይነት መድሓኒት ወሲዱ/ዳ (ኾላይ ኣብ ሆስፒታል እንዳሃለወ/ት ዝወሰዶ/ቶ እንተሃልዩ)?

☐ እወ ወሲዱ/ዳ ☐ ኣይወሰደን/ትን

8. እንታይ ዓይነት መድሓኒታት ወሲዱ/ዳ ነይሩ/ራ? **ሓበሬታ ንሓታታይ፡ ቀፂሉ ዘሎ ቅጥዒ ተጠቂሞም ይምልእዎ**

ኮድ እቲ መድሓኒት	መድሓኒት	መድሓኒቱ ዝተወሰደሉ መንገዲ	መድሓኒት ዝአዘዘ አካል	እቲ መድሓኒት ዝተገዝአሉ (ዝተረኸበሉ)
	ሽም እቲ መድሓኒት ዝፍለጥ እተኾይኑ ይፅሓፍ እንተዘይኾይኑ ድማ በቲ ሓበሬታ ዝወሃበካ (ካብቲ መልሲ ወሃቢ) ናይ እቲ መድሓኒት ምድቡ ይፅሓፍ)	1. ብኣፍ<br>2. ብመርፌእ<br>3. ኻሊእ እንተኾይኑ ይግለፅ	1. ባዕለይ<br>2. አባል ስድራ<br>3. ዓርኪ/ጎረቤት<br>4. ሓኪም/ነርስ<br>5. ናይ ባህላዊ ሓኪም<br>6. በዓል ሞያ መድሓኒት<br>7. ካልኦት (ይግለፅዎም)	1. ካብ ስድራ ቤት፣ ዓርኪ፣ ጎረቤት<br>2. ካብ ናይ ህዝቢ ሆስፒታል<br>3. ካብ ናይ ህዝቢ ጥዕና ጣብያ<br>4. ካብ ናይ ግሊ ጥዕና ተቋማት<br>5. ናይ ባህላዊ ሓካይም<br>6. ናይ ግሊ ቤት መድሓኒት<br>7. ካብ ቅድሚ ሐዚ ዝተሓከሙ ሰባት ዝተረፈ መድሓኒት<br>8. ካብ ቤት ሹቕ<br>9. ካብ ካሊእ (ይዘርዝርዎም)
መድሓኒት 1				
መድሓኒት 2				
መድሓኒት 3				
መድሓኒት 4				

መድሓኒት 5				
መድሓኒት 6				

9. እቲ ዝሓመመ ሰብ እቲ ዝተአዘዘሉ ኩሉ መድሓኒት ብኣግባቡ ተጠቒምዎ'ዶ

☐ እወ ተጠቒሙ ☐ አይተጠቐሞን

ሓበሬታ:- እንድሕር መልሱ "ተጠቒሙ" ኮይኑ ቀጥታ ናብ ተ.ቁ 11 ይኺዱ

10. እንድሕርደአ እቲ መድሓኒት ሙሉ ብሙሉእ ዘይተጠቀሞ/ተጠቂሙእን ተቋሪፅዎ ምኽንያቱ ንምቁራፅ ወይ ንዘይምጥቃም ካብዞም ዝስዕቡ መኒኦም እዮም?

- ☐ ጥዕንኡ/ኣ ዝተመሓየሸ ኮይኑ ስለተሰመዮ/ዓ
- ☐ ናይ ኩሎም መድሓኒታት አድላይነቱ ዘይምእማን
- ☐ ናይቲ መድሓኒት ፈዋሲነቱ ስለዘይአመነሉ/ትሉ
- ☐ ንቀፃሊ እንተሓሚሚዎ ንኽጥቀሙሉ ከቐምጥዎስለዝደልዩ
- ☐ ናይቲ መድሓኒት ናይ ጎናዊ ችግር ስለዘለዎ
- ☐ ካብ አባል እቲ ስድራ ቤት ሓደ አደላይነት እቲ መድሓኒት ስለዘይአመነሉ/ትሉ
- ☐ ዝኾነ ሰብ እቲ መድሓኒት ከይወሰዶ ስለ ዝመኸሮ
- ☐ ሕሙም ሰብ ቅድሚኡ እተ መድሓኒት ስለተአነፎ/ፋ
- ☐ ካብቲ አባል ስድራ ቤት ሓደ ሰብ ኻሊእ ናይ ሕክምና ዘዴ ስለዝመረፀ
- ☐ ኻሊእ ምኽንያት ተሃልዩ ይዘርዝሩ____________________
